# Fast structure similarity searches among protein models: efficient clustering of protein fragments

**DOI:** 10.1186/1748-7188-7-16

**Published:** 2012-05-29

**Authors:** Federico Fogolari, Alessandra Corazza, Paolo Viglino, Gennaro Esposito

**Affiliations:** 1 Department of Biomedical Sciences and Technologies, University of Udine Piazzale Kolbe 4, 33100 Udine - Italy and Istituto Nazionale Biostrutture e Biosistemi Viale Medaglie d'Oro 305, 00136 Roma, Italy

## Abstract

**Background:**

For many predictive applications a large number of models is generated and later clustered in subsets based on structure similarity. In most clustering algorithms an all-vs-all root mean square deviation (RMSD) comparison is performed. Most of the time is typically spent on comparison of non-similar structures. For sets with more than, say, 10,000 models this procedure is very time-consuming and alternative faster algorithms, restricting comparisons only to most similar structures would be useful.

**Results:**

We exploit the inverse triangle inequality on the RMSD between two structures given the RMSDs with a third structure. The lower bound on RMSD may be used, when restricting the search of similarity to a reasonably low RMSD threshold value, to speed up similarity searches significantly. Tests are performed on large sets of decoys which are widely used as test cases for predictive methods, with a speed-up of up to 100 times with respect to all-vs-all comparison depending on the set and parameters used. Sample applications are shown.

**Conclusions:**

The algorithm presented here allows fast comparison of large data sets of structures with limited memory requirements. As an example of application we present clustering of more than 100000 fragments of length 5 from the top500H dataset into few hundred representative fragments. A more realistic scenario is provided by the search of similarity within the very large decoy sets used for the tests. Other applications regard filtering nearly-indentical conformation in selected CASP9 datasets and clustering molecular dynamics snapshots.

**Availability:**

A linux executable and a Perl script with examples are given in the supplementary material (Additional file 1). The source code is available upon request from the authors.

## Background

Computational predictions and simulations of biological systems entail a wide variety of processes and length and time scales. Going down from ecological systems, to organisms, organs and cells and subcellular components, the lowest level description of biological systems is in terms of single molecules and atoms [1]. At this level, the structure and dynamics of biomolecules and biocomplexes are of utmost importance in determining their function, whose knowledge and elucidation is the ultimate goal of structural biology. Experimental methods for structural characterization of biomolecules are often too slow or have limitations in targets and resolution that cannot be overcome. For these reasons one often resorts to computational predictions or simulations.

A common feature of computational methods is that they generate a large number, typically in the range of thousands, of molecular models which are meant as samples of the large conformational space of a molecule or of a complex.

As a consequence, clustering of different conformations of the same molecular structure is a frequently performed task which allows on the one hand to reduce the number of conformations to be subjected to further analysis, and on the other hand to choose the most representative conformations among many [2]. Clustering is mostly performed based on pairwise distance, see e.g. the GROMACS package manual for some widely used methods (URL: http://www.gromacs.org). In many cases a dissimilarity criterion is used instead of a similarity criterion (see e.g. for a general discussion [3,4]). These issues are well illustrated in the fields of protein structure predictions and molecular dynamics simulations.

For instance, prediction of protein structure is typically accomplished in two steps: first a large number of plausible models (possibly including near-native models) is generated and afterwards models are scored and ranked [5]. The latter step often considers each model as representative of an ensemble of similar conformations and some kind of weighted average is performed to pick up the best model (see e. g. [6-9]). For what concerns proteins the field of structural predictions has been largely explored in the last decades and the world-wide experiment CASP (Critical Assessment of Structural Predictions) has set standards for evaluation and has provided extensive evaluation of methods and approaches [10]. Since 2006, among other prediction categories, the model Quality Assessment (QA) category has been introduced where predictors are asked to evaluate the quality of the many models (in the range of hundreds) proposed by servers for each target sequence. In recent rounds of CASP consensus methods have consistently scored better than single structure methods. Consensus may be taken on the score given by different model quality assessment programs or on the similarity of the models within the ensemble itself. Actually in the last CASP8 and CASP9 experiments the top performing model quality estimators were those using clustering/consensus methods on the ensemble of deposited models. Using the similarity among different predictive models was recognized earlier as a significant improvement factor for assessing the quality of a model [11]. Since then methods have known continuous evolution up to the recent remarkable achievements in blind assessment experiment by the programs QmeanClust [12,13], 3D-Jury [14], Pcons [15], ModFoldClust [16] and others [9]. In these methods the similarity among predictive models, as well as scores obtained sometimes by different, possibly independent, sources, is used in different ways tailored on the specific model quality assessment method used. In some, but not all the methods, only similarities above a given threshold are used [14]. The reader is referred to the original literature for details.

Somewhat surprisingly, however, a naive consensus method ranking the models based on the average similarity with other models in the ensemble was found to perform like the best consensus model quality estimators [9] as earlier suggested by Elofsson and coworkers [11]. Structural comparisons appear therefore very important for proper choice or scoring of predictive models.

Similarly to predictive tasks, in molecular dynamics simulations typically many conformations are generated, in the range of thousands, by taking snapshots of the trajectory at given time intervals. Here clustering of different conformations of the same molecular structure is performed to reduce the number of conformations to be subjected to further analysis (e.g. docking simulations), or to choose the most representative conformations among many [2].

The measures of similarity that are typically used for proteins have been set in the context of the CASP experiment. The most straightforward measure of (dis)similarity is the root mean square deviation (RMSD) of corresponding atoms after optimal superposition of two molecular structures. This measure requires only the definition of the set of atoms to be superimposed and the set of atoms on which to compute the RMSD. The RMSD is considered sometimes less sensitive than other measures because well modeled parts of the protein will be not represented by the RMSD dominated by large deviations and, viceversa, large deviations in small regions will not contribute significantly to the RMSD computed from averaging over a typically much larger set of atoms.

Several other measures of similarity have been proposed to give a representation of similarity which reflects the fraction of well modeled residues. In this respect MaxSub [17], GDT_TS [18] and TM-score [19] are considered more appropriate than the RMSD. The details and motivations of these similarity scores are reported in the original papers and they will not be repeated here. Suffice it to say that these methods assign a score based on the maximum number of residues that can be superimposed at one or more given distance threshold. It is worth to remind that these scores are assigned through iterative algorithms that require by far more computations than a single RMSD computation. For this reason we will consider here the widely used definition of distance as the RMSD between corresponding C_α _(CA) atomic positions after optimal superposition. However, as long as a dissimilarity measure is a metric the methodology reported hereafter applies.

Once a distance definition has been chosen, in the consensus procedures or representative model selections discussed above, the all-vs-all pairwise similarity computations are straightforward to implement, but the time required to analyse a set of, say, 10000 structures may become exceedingly large, requiring approximately 50 × 10^6 ^comparisons.

There are two issues to consider with all-vs-all comparisons. The first is the quadratic number of comparisons and the second is that RMSD computation is time consuming itself.

The latter aspect has been investigated and, compared to earlier methods of RMSD calculation [20,21], significantly faster solutions which avoid finding the optimal rotation matrix, have been proposed based on quaternion formalism [22].

An approximated solution to the all-vs-all comparison problem has been proposed by Li and Zhou [23], who avoid finding the $\frac{N\left(N-1\right)}{2}$ minimum RMSDs altogether, but rather choose randomly a reference structure, superpose only once all structures on the reference one (using *N *- 1 rototranslations) and compute the RMSD without any further rototranslation. The authors showed that the choice of reference structure is almost ininfluential, at least on their datasets, and that the RMSD computed in this way furnishes a good approximation to the minimum RMSD.

Here we improve the efficiency of all-vs-all comparisons by considering that often only good similarities are searched for. If a threshold RMSD value is set reasonably low, compared to the average random RMSD in the set, many comparisons can be avoided by using the inverse triangular inequality satisfied by the RMSD. We exploit this fact and we show that a significant speed-up is obtained on large decoy sets. An application to clustering of a very large number of fragments to a representative set is presented as an example. A more realistic scenario where different models for the same protein (resulting from structural predictions or molecular dynamics simulations) is provided by the search of similarity within very large decoy sets. Other applications like filtering nearly identical structures in a set or clustering molecular dynamics snapshots are shown as examples.

## 1 Methods

Given a set of N decoys a straighforward similarity search would require $\frac{N\;\times \;\left(N-1\right)}{2}$ structural comparisons. After the first structure is compared with all the others, however, the corresponding set of pairwise RMSDs is available. We use the set of pairwise comparisons with the common reference structure to exclude from comparison those pairs of structures whose RMSD is surely above the threshold.

Let us define ${\mathrm{RMSD}}_{\mathrm{ij}}^{\mathrm{opt}}$ and *RMSD_ij _*as the RMSD between structures *i *and *j*, after optimal superposition and with no optimal superposition, respectively. It has been shown by Edwards et al. [24] and by Steipe and Kaindl [25,26] that *RMSD^opt ^*is a metric on the space of the classes of equivalent structures (i. e. structures that can be superimposed exactly by a rototranslation). As a consequence both the triangle inequality $\left(RMSD_{ij}^{opt}\;<=\;RMSD_{ik}^{opt}\;+\;RMSD_{kj}^{opt}\right)$ and the inverse triangle inequality $\left(RMSD_{ij}^{opt}\;>=\;|RMSD_{ik}^{opt}\;-\;RMSD_{kj}^{opt}|\;\right)$ hold. Note that the metric properties of *RMSD^opt ^*are not trivial as for RMSD because a different rototranslation is in principle implied by each *RMSD^opt^*. As a consequence of RMSD*^opt ^*being a metric, the following inverse triangle inequality holds:

(1) $$RMSD_{ij}^{opt}\;\geq \;|RMSD_{1i}^{ipt}\;-\;RMSD_{1j}^{opt}|$$

(In the appendix we provide a brief demonstration of the inverse triangle inequality for *RMSD^opt^*). Based on the inverse triangle inequality, if we have computed $RMSD_{1i}^{opt}$ with *i *= 2, .., *N *it is possible to exclude from further comparison all pairs *i *and *j *for which $\left|RMSD_{1i}^{opt}\;-\;RMSD_{1j}^{opt}\right|$ is larger than the chosen threshold *t*. By using this principle similarity searches may be sped up significantly.

Let us have an ensemble of *N *structures to be searched for structural similarity and let us assume that the distribution of pairwise RMSDs within the ensemble is *f*(*x*) (*f*(*x*) has been found by us in many decoys sets to be close to a normal distribution).

The algorithm we will describe iterates over steps requiring first a one-vs-all comparison (*N *- 1 comparisons when we have *N *structures) and then using the list of RMSDs to avoid unnecessary comparisons. In the latter task the number of comparisons depends, obviously, on the RMSDs distribution, but also on the use we make of the already computed RMSDs.

If the list of RMSDs is sorted, the structures corresponding to lowest RMSD will be the more distant from other structures and the inverse triangular inequality will make most comparisons unnecessary. On the other hand as we consider structures whose RMSD is in the most populated region of the distribution there will be many other structures with similar RMSD and the inverse triangular inequality will make many fewer comparison unnecessary.

It will be therefore more efficient to use the inverse triangular inequality only on the structures displaying an RMSD below a certain value *s*.

The number of comparisons *N_cmp _*involved at the step when *N *structures have not been compared yet, will be the sum of the (*N *- 1) comparisons needed to generate the list of (*N *- 1) RMSDs and the number of comparisons that make use, through the inverse triangular inequality, of the RMSD list computed:

(2) $$N_{cmp}\;=\;\;\left(N\;-\;1\right)\;+\;\left(N\;-\;1\right)\left(N\;-\;2\right)\;\int_{0}^{s}f\left(x\right)\;\left(\int_{x}^{x+t}f\left(x^{\prime }\right)dx^{\prime }\right)\;dx$$

where *s *is the RMSD where we stop using the inverse triangular inequality on the already computed RMSDs and we move to the next step calculating RMSDs for a single structure and using the newly computed RMSDs.

After the comparisons have been made the number of structures whose RMSD below threshold *t *with all other structures have been found (*N_done_*) will be:

(3) $$N_{done}\;=\;1\;+\;\left(N\;-\;1\right)\;\int_{0}^{s}f\left(x\right)dx$$

At each step *s *should maximize the ratio $\frac{N_{done}}{N_{cmp}}$ based on the observed distribution of RMSDs. In practice the value *s*, i.e. the maximum RMSD whose corresponding structure is compared to all others using the inverse triangular inequality, is chosen when the ratio $\frac{N_{done}}{N_{cmp}}$, which is computed while the comparisons are done, reaches a maximum.

Hereafter the implementation described above is reported in C-like pseudocode:

/* Initialization */

for(i = 0; i < n_structures; i++)

   done[i] = 0;

/* Iterations */

for(i1 = 0; i1 < n_structures; i1++)

{

   n_cmp = 0;

   k = 0;

   for(i2 = i1 + 1; i2 < n_structures; i2++)

   {

      if(!done[i2])

      {

         index[k] = i2

         rmsd[k] = RMSD(struct i1, struct i2)

         n_cmp = n_cmp + 1

         if(rmsd <= threshold )

            output(i1, i2, rmsd)

         k = k + 1

      }

   }

   done[i1] = 1

   n_left = k

   index_rmsd <- (index, rmsd)

   sort index_rmsd by rmsd

   ratio = 0

   for(j = 0; ((j+1)/n_cmp) > ratio && j<n_left; j++)

   {

      ratio = ((j+1)/n_cmp)

      for(k=j+1; k<n_left; k++)

         if ((index_rmsd.rmsd[k] - index_rmsd.rmsd[j]) <= threshold)

         {

            rmsd = RMSD(index_rmsd.index[k], index_rmsd.index[j])

            n_cmp = n_cmp + 1

            if(rmsd <= threshold)

               output(index _rmsd.index[k], index_rmsd.index[j], rmsd)

         }

      done[index_rmsd[j].index] = 1

   }

}

Better schemes keeping track of all the already computed RMSDs would require larger memory. The algorithm has been tested on decoy sets for small proteins downloaded from the Decoys'r'us database [27], representing a realistic step in a clustering scenario.

## 2 Results and discussion

### 2.1 Tests on decoy sets

The effect of the choice of the threshold has been tested on the relatively small 4state_reduced decoy datasets [28] which include on average 665 decoys for each target protein.

RMSD computation has been performed on all C*_α _*carbons. Four reasonable threshold values have been tested (2.4, 2.8, 3.2, 3.6 Å) with the results reported in Table 1. The effect of the RMSD threshold on the number of computations required is apparent at the lower end of the range explored where 45% of the computations are avoided. As expected the smaller the threshold the lower number of computations are required. This suggests that RMSD computations could be applied iteratively by clustering structures at increasing RMSD threshold value. This idea is applied hereafter.

**Table 1:** Number of RMSD computations for the 4state_reduced decoy dataset with varying RMSD threshold.

Decoy set	t = 2.4 Å	t = 2.8 Å	t = 3.2 Å	t = 3.6 Å	$\frac{N\left(N-1\right)}{2}$
1ctf	87,219	103,780	122,095	140,094	198,765
1r69	130,510	150,415	171,407	189,659	228,150
1sn3	106,523	124,587	147,101	174,527	217,470
2cro	183,135	206,133	189,277	221,219	227,475
3icb	130,653	119,774	139,425	176,184	213,531
4pti	106,581	131,345	175,432	191,091	236,328
4rxn	95,306	117,515	143,481	190,997	228,826

Total	839,927	953,549	1,088,220	1,283,751	1,550,545

The method has been tested on the semfold decoy datasets which include on average 12900 decoys and in one case more than 20000 decoys [29].

The reduction in the number of computations required compared to the all-vs-all scheme, i. e. $\frac{N\;\times \;\;\left(N\;-\;1\right)}{2},$ is significant and makes on average 87% of the comparisons unnecessary, proving its usefulness (Table 2). Besides the speed-up in computation time, the scheme presented above has the further advantage of requiring only memory proportional to *N *for storing the list of structure files and RMSDs of one structure with all the remaining ones, as evident in the pseudo-code detailed above.

**Table 2:** Number of RMSD computations for the semfold decoy dataset.

Decoy set	This work	$\frac{N\left(N-1\right)}{2}$	ratio
1ctf	11,753,426	64,997,101	0.18
1e68	9,039,397	64,541,841	0.14
1eh2	7,332,361	65,453,961	0.11
1khm	22,047,014	222,193,740	0.10
1nkl	7,966,008	67,995,291	0.12
1pgb	13,465,834	63,636,121	0.21

Total	71,604,689	548,818,055	0.13

For this table the RMSD threshold was 3.0 Å.

**Figure 1:**
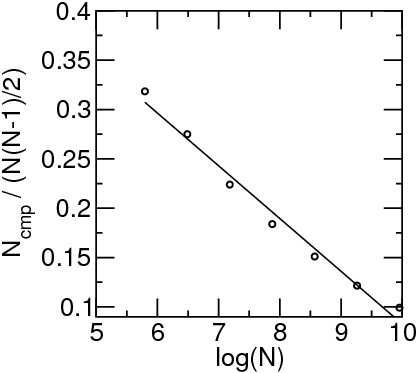
**Ratio of the number of RMSD computations performed over *N*(*N *- 1)/2 versus the logarithm of the number of structures in subsets from the semfold 1khm decoy set**.

Comparing the results reported in Tables 1 and 2 there appears to be an effect of the ensemble size *N *on the ratio of the actual computations over *N*(*N *- 1)/2. This effect is not apparent from equations 2 and 3 because the integration upper limit s depends implicitly on *N*. Assuming heuristically that the RMSDs are distributed according to a simple sine law, equations 2 and 3 can be integrated and the ratio is found to decrease with the size of the ensemble. In order to test this suggestion, for the largest set (semfold 1khm set, 22000 structures) we considered sets with 1/2, 1/4, 1/8, 1/16, 1/32, 1/64 of the structures and plotted the ratio of computations over *N*(*N *- 1)/2 against *log*(*N*). The dependence is almost linear in the range considered (Figure 1).

### 2.2 Clustering fragments from the top500H dataset

As expected the above tests show that for lower RMSDs application of the inverse triangular inequality is very efficient in reducing the number of comparisons to be performed. The idea is exploited here to cluster short protein fragments.

Fifirst RMSDs below a very low threshold are computed. The computations will be many less than $\frac{N\;\times \;\;\left(N\;-\;1\right)}{2},$ because the inverse triangular inequality is used in the best way at low thresholds.

Based on the computed RMSDs fragments are clustered and each fragment is weighted based on the proximity of similar fragments. Among the many schemes available we have assigned a weight according to the following equation:

$$w_{i}\;=\;\sum w_{j}^{0}\;\text{cos}\;\left(\pi \;\frac{RMSD}{2t}\right)$$

where $w_{j}^{0}$ is the weight before clustering (i.e. 1 in the first clustering and greater or equal to 1 in subsequent clusterings) and *t *is the threshold RMSD chosen. This scheme is similar to others suggested for weighting similar structures [30] or for choosing the most representative fragment among similar ones [7,8]. The list of fragments is then sorted by the weight and for each fragment, starting with the one with larger weight, all other fragments with RMSD less than threshold from the reference one are clustered together and represented by the reference one.

The RMSD threshold is increased and the procedure repeated until a single cluster is found.

As an example we have taken the dataset obtained by considering all continuous five residues fragments from the proteins in the top500H dataset [31] which includes 500 curated non redundant protein structures obtained by X-ray crystallography with resolution better than 1.8 Å and with few deviations from ideal geometry.

We have chosen a length of five following Micheletti et al. [30] who showed that a small dataset of five-residues fragments is able to represent accurately all five-residues fragments. Many of these fragments are nearly identical because of secondary structure elements and therefore the task of clustering residues should involve either a filtering or a large number of comparisons. The number of fragments is 107184 which implies, for an all-vs-all comparison ca. 5.7 billion RMSDs computations.

Our algorithm was able to cluster at various thresholds the fragments in a couple of hours on a laptop, with minimal memory requirements.

The results are listed in Table 3 where the number of starting fragments and the number of representative fragments is reported together with the number of RMSD computations performed and the number of all-vs-all computations.

**Table 3:** Fragment clustering.

RMSD threshold (Å)	**N. struct**.	This work	$\frac{N\left(N-1\right)}{2}$	ratio	**Rep. Struct**.
0.05	107,184	57,978,627	5,744,151,336	0.0100	79,994
0.1	79,994	28,610,140	3,199,480,021	0.0089	47,502
0.2	47,502	19,089,084	1,128,196,251	0.0169	13,066
0.4	13,066	4,479,481	85,353,645	0.0525	1,853
0.8	1,853	393,824	1,715,878	0.2295	131

Total	107,184	110,551,156	5,744,151,336	0.0193	

Columns report the threshold RMSD chosen for clustering, the number of starting fragments, the number of RMSD computations done, the number of computations in an all-vs-all comparison, the number of representative fragments, used as starting fragments at the next iteration. CA atoms have been used for superposition.

**Figure 2:**
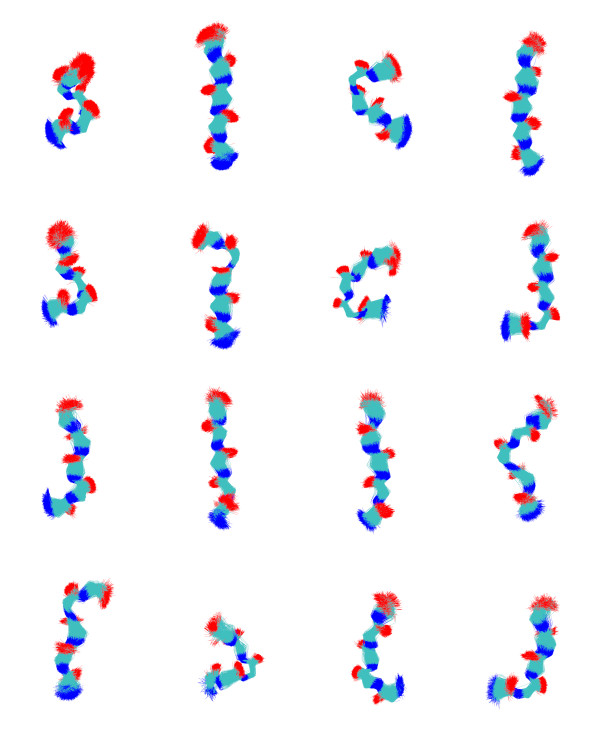
**Fragments superposed on the representative fragment for the 16 cluster populated with more than 1% of the whole dataset**.

In table 4 similar figures are reported with the difference that superposition is performed on atoms N,CA,C,O. The structures grouped in the 16 clusters that include more than 1% of the whole fragment dataset have been superimposed to the representative structure and they are displayed in Figure 2. Typical conformations of *α*-helices and *β*-strands are found in the first and second group, respectively, as expected. Other groups are associated with tight turns, with *α*-helix and *β*-strand terminal motifs and with different additional *β*-strand conformations.

**Table 4:** Fragment clustering on backbone.

RMSD threshold (Å)	**N. struct**.	This work	$\frac{N\left(N-1\right)}{2}$	ratio	**Rep. Struct**.
0.05	107,184	53,793,796	5,744,151,336	0.0094	105,299
0.1	105,299	124,506,221	5,543,887,051	0.0225	87,195
0.2	87,195	57,001,567	3,801,440,415	0.0150	63,829
0.4	63,829	65,723,340	2,037,038,706	0.0322	21,637
0.8	21,637	33,375,313	234,069,066	0.1426	2,445
1.6	2,445	2,586,436	2,987,790	0.8657	33
3.2	33	528	528	1.0000	2

Total	107,184	336,987,202	5,744,151,336	0.0587	

Columns report the threshold RMSD chosen for clustering, the number of starting fragments, the number of RMSD computations done, the number of computations in an all-vs-all comparison, the number of representative fragments, used as starting fragments at the next iteration. Atoms N, CA, C, O have been used for superposition.

From Tables 3 and 4 it is immediately seen how effective is the fast structural similarity search proposed in this work compared to all-vs-all comparisons. The number of actual computations is just 2% and 6% of that implied by all-vs-all comparisons for data in Tables 3 and 4, respectively.

Overall these results confirm the reliability of the methodology whose implementation is made possible by the fast computation of structural similarities.

### 2.3 Filtering predictive models for model quality assessment

The tests performed on decoy sets are representative of a possible clustering scenario in the context of most representative model selection. In the context of the CASP esperiment the models available are in the range of few hudreds and therefore the advantage of our method is limited. We address here another application consisting in filtering most similar models before applying consensus methods. Consensus methods rely on the independence of the predictive models. Although in principle it would be desirable to define a distance based on most used similarity measures like MaxSub [17], GDT_TS [18] or TM-score [19] it is difficult to enforce in the latter measures the metric properties (in particular the triangular property) that are needed for the present method to work. For this reason we used the RMSD as pairwise distance. Since the models may be of different, even non overlapping, lengths we normalized the RMSD on the number of the aligned residues according to simple scaling proposed by Carugo and Pongor [32]:

$$RMSD_{100}\;=\;\frac{RMSD}{1\;+\;\frac{1}{2}\text{log}\left(\frac{N}{100}\right)}$$

where *RMSD*_100 _is the estimated RMSD when 100 residues are aligned and *N *is the number of aligned residues. When *N *is less than 14 the *RMSD *is set to a maximum value.

We chose all server predictions for FM (free modeling) targets in the recent CASP9 experiment (i.e. T0529, T0531, T0534, T0537, T0544, T0547, T0550, T0553, T0555, T0561, T0571, T0578, T0581, T0604, T0608, T0616, T0618, T0621, T0624, T0629, T0637 and T0639). We assume that FM models that display a normalized pairwise RMSD below 1.0 Å are not independent and therefore only one representative model should be considered. In this way all nearly identical models are identified. In the sets considered there are many models (deposited by the same predictors) that are identical.

The effectiveness of the procedure may be judged by considering the number of comparisons performed (115399) versus those to be performed in an all-vs-all comparison (1040520) which amounts to a ratio of 0.11. The procedure allows to remove on average 40 predictive models that have a nearly identical model in each set.

### 2.4 Clustering molecular dynamics snapshots

The analysis of molecular dynamics trajectories often involves the comparison and clustering of thousands of snapshots. Clustering is mostly based on the analysis of pairwise RMSDs. When only closest similar conformations are to be grouped together the method proposed here strongly reduces the number of comparisons to be performed.

In the following two illustrative applications of the method are given.

We consider the decoy set vhp_mcmd [33], a decoy set containing alternative conformations for the villin headpiece obtained from snapshots of long molecular dynamics runs in explicit solvent starting from five different conformations, including the native one. The decoy set includes 6255 conformations. In order to choose the most representative ones we consider similarities at 1.0 Å threshold. The search at this similarity level is performed with 1717823 comparison, i.e. 0.088 times the comparisons required by a straightforward all-vs-all comparison (6255 × 3127). Clustering of structures based on the obtained similarities allows to identify for each of the five simulations a representative structure. Among these the largest cluster (245 out of 651 structures) is found for the simulation starting from the native structure, which highlights the lesser conformational dispersion for simulations starting from the native structure with respect to those starting from non-native structures.

We consider as another application the analysis of the simulation of cis-trans isomerization of Pro 32 in *β*2-microglobulin that drives a switch in the hydrogen bonding network of residues Arg 3, Thr 4, His 31, Pro 32, His 84 and Thr 86 [34]. We consider the pairwise RMSD after superposition of all the N, CA, C and O atoms of the above six residues. We set the threshold at 0.4 Å to spot significantly different conformations. The number of comparison performed is 0.25 of those required by an all-vs-all comparison. The first two most representative clustered conformations are relative to the native cis Pro 32 conformation and non-native trans Pro 32 conformation, thus allowing selection of most representative conformations at the selected region.

## 3 Conclusions

The proposed method for fast structural similarity search below a given distance threshold makes use of the inverse triangular disequality in order to avoid unnecessary comparisons.

The applications reported here shows that the method is able to save many comparisons whenever the distance threshold is set much smaller than the average distance between structures in the set.

The advantage of the method compared to all-vs-all comparisons is more and more evident with larger datasets.

The method seems therefore mostly suited to calculate fastly similarities among large ensembles of conformers, e.g. those obtained by predictive softwares like Rosetta [35] where thousands of models are typically generated, or for large structural database analysis. In this respect the first three applications described in the Results section appear particularly suited for the method. Analysis of molecular dynamics snapshot is also an important field of application. The only drawback of the method is that the threshold distance between conformations must be much lower than the average distance for the method to be efficient. In this respect the comparison of conformations of subsystems (that display conformational mobility) or comparison at a very low threshold distance (aiming at identifying nearly identical conformers) appear to be the situation of choice for the application of the method, as shown in the examples of the Results section.

The main limitation of the method is that it relies on the use of the metric properties of the dissimilarity measure. For this reason it is not evident how to extend the application in order to make use of useful similarities measures like MaxSub [17], GDT_TS [18] and TM-score [19]. Turning the latter measures into proper metrics is a prerequisite for using the method in predictive contexts like that set up in the CASP experiment. On the other hand it is apparent that the method may be used also in other contexts whenever a metric can be defined and similarities at low distances (compared to the average pairwise distance) are sought.

## 4 Availability

A reference program for Linux, using, with minor modifications, the RMSD calculation routines of Theobald [22], is available as supplementary material (Additional file 1). The source code is available upon request from the authors. A Perl script implementing the algorithm is provided with a subroutine *dist()* that wraps any external measure of distance.

## 5 Competing interests

The authors declare that they have no competing interests.

## 6 Authors' contribution

FF conceived the study and implemented the algorithm, AC, PV and GE contributed design of the study and the tests. All authors read and approved the manuscript.

## Appendix

Let us consider two structures *i *and *j *which have been optimally superposed on structure 1, so that $RMSD_{1i}^{opt}$ and $RMSD_{1j}^{opt}$ are known. We look for a lower bound on $RMSD_{1j}^{opt}$ based on $RMSD_{1i}^{opt}$ and $RMSD_{1j}^{opt}$.

First, we traslate all structures in such a way that the center of geometry of atoms to be superposed is the same for all structures. Second we write the ${\mathrm{RMSD}}_{\mathrm{ij}}^{\mathrm{opt}}$:

(4) $${\left({\mathrm{RMSD}}_{\mathrm{ij}}^{\mathrm{opt}}\right)}^{2}\;\;=\;\;\frac{\sum_{k=1,N}||{\vec{r}}_{\mathrm{ik}}\;-\;R_{j\to i}^{\mathrm{opt}}{\vec{r}}_{jk}{\left|\right|}^{2}}{N}$$

(5) $$=\;\frac{\sum_{k=1,N}||\;R_{i\to j}^{opt}{\vec{r}}_{ik}\;-\;{\vec{r}}_{jk}|{|}^{2}}{N}$$

where *r_ik _*and *r_jk _*are the vectors of atom *k *in structure *i *and *j*, respectively, and $R_{i\to j}^{opt}$ is the rotation that superposes optimally, i.e. with the least RMSD, structure *i *onto structure *j*.

If we arrange the 3 × *N *atomic coordinates *r_ik _*in a single vector *v_i _*and build a 3*N *× 3*N *block diagonal matrix $\bar{R}$ by repeating the 3 × 3 rotation matrix *R N *times along the diagonal, we can simplify the above notation:

(6) $$\sqrt{N}\;\times \;RMSD_{ij}^{opt}\;=\;||{\vec{v}}_{i}\;-\;{\bar{R}}_{j\to i}^{opt}{\vec{v}}_{j}||$$

(7) $$=\;||{\vec{v}}_{j}\;-\;{\bar{R}}_{i\to j}^{opt}{\vec{v}}_{i}||$$

Let us define $v_{1}^{{}^{\prime }}$ as ${\bar{R}}_{i\to j}^{opt}{\bar{R}}_{1\to j}^{opt}{\vec{v}}_{1}.$ Note that $\sqrt{N}RMSD_{1j}^{opt}\;=\;||{\bar{R}}_{1\to j}^{opt}{\vec{v}}_{1}\;-\;{\vec{v}}_{j}||\;=\;||{\bar{R}}_{i\to j}^{opt}\left({\bar{R}}_{1\to j}^{opt}{\vec{v}}_{1}\;-\;{\vec{v}}_{j}\right)||$ because the norm is invariant under rotations.

Now we can rewrite equation (6) and derive a lower bound for ${\mathrm{RMSD}}_{\mathrm{ij}}^{\mathrm{opt}}$ based on $RMSD_{i1}^{opt}$ and $RMSD_{j1}^{opt}.$ In the next equation we assume that $RMSD_{i1}^{opt\;}\;\geq \;RMSD_{j1}^{opt}$ without loss of generality.

(8) $$\sqrt{N}\;\times \;RMSD_{ij}^{opt}\;\;=\;\;||\left({\vec{v}}_{i}\;-\;{\bar{R}}_{j\to i}^{opt}{\vec{v}}_{j}\right)||$$

(9) $$=\;\;||\left({\vec{v}}_{i}\;-\;{\vec{v}}_{1}^{{}^{\prime }}\right)\;+\;\left({\vec{v}}_{1}^{{}^{\prime }}\;-\;{\bar{R}}_{j\to i}^{opt}{\vec{v}}_{j}\right)||$$

The inverse triangle inequality applies to euclidean distance:

(10) $$\left||\left({\vec{v}}_{i}\;-\;{\vec{v}}_{1}^{{}^{\prime }}\right)\;+\;\left({\vec{v}}_{1}^{{}^{\prime }}\;-\;{\bar{R}}_{j\to i}^{opt}\;{\vec{v}}_{j}\right)||\;\;\geq \;\;|||{\vec{v}}_{i}\;-\;{\vec{v}}_{1}^{{}^{\prime }}||\;-\;||{\vec{v}}_{1}^{{}^{\prime }}\;-\;{\bar{R}}_{j\to i}^{opt}{\vec{v}}_{j}||\right|$$

By rendering explicit the second term in the right-hand member of the inequality we get:

(11) $$\left|||{\vec{v}}_{i}\;-\;{\vec{v}}_{1}^{{}^{\prime }}||\;-\;||{\vec{v}}_{1}^{\prime }\;-\;{\bar{R}}_{j\to i}^{opt}{\vec{v}}_{j}|||\;\;=\;\;|||{\vec{v}}_{i}\;-\;{\vec{v}}_{1}^{{}^{\prime }}||\;-\;||{\bar{R}}_{j\to i}^{opt}\left({\bar{R}}_{1\to i}^{opt}{\vec{v}}_{1}\;-\;{\vec{v}}_{j}\right)||\right|$$

(12) $$=\;\;\;|\sqrt{N}RMSD_{1^{\prime }i}\;-\;\sqrt{N}RMSD_{1j}^{opt}|$$

Note that in the latter equation the first term is the RMSD between rototraslated structure 1 and structure *i *with no optimal superposition. Since it is assumed that $RMSD_{i1}^{opt\;}\;\geq \;RMSD_{j1}^{opt}$ the following inequality holds:

$$\left|\sqrt{N}RMSD_{1^{\prime }i}\;-\;\sqrt{N}RMSD_{1j}^{opt}|\;\geq \;\sqrt{N}|RMSD_{1i}^{opt}\;-\;RMSD_{1j}^{opt}\right|$$

As a consequence of the above equations the inverse triangular inequality holds:

(13) $$RMSD_{ij}^{opt}\;\geq \;|RMSD_{1i}^{opt}\;-\;RMSD_{1j}^{opt}|$$

## Supplementary Material

Additional file 1 **Zipped le with the Linux executable and example files**. Click here for file
